# Itaconate, Arginine, and Gamma-Aminobutyric Acid: A Host Metabolite Triad Protective Against Mycobacterial Infection

**DOI:** 10.3389/fimmu.2022.832015

**Published:** 2022-02-04

**Authors:** Jin Kyung Kim, Eun-Jin Park, Eun-Kyeong Jo

**Affiliations:** ^1^ Department of Microbiology, Chungnam National University College of Medicine, Daejeon, South Korea; ^2^ Infection Control Convergence Research Center, Chungnam National University College of Medicine, Daejeon, South Korea

**Keywords:** itaconate, arginine, GABA, host defense, *Mycobacterium tuberculosis*, innate immunity

## Abstract

Immune metabolic regulation shapes the host-pathogen interaction during infection with *Mycobacterium tuberculosis* (Mtb), the pathogen of human tuberculosis (TB). Several immunometabolites generated by metabolic remodeling in macrophages are implicated in innate immune protection against Mtb infection by fine-tuning defensive pathways. Itaconate, produced by the mitochondrial enzyme immunoresponsive gene 1 (IRG1), has antimicrobial and anti-inflammatory effects, restricting intracellular mycobacterial growth. L-arginine, a component of the urea cycle, is critical for the synthesis of nitric oxide (NO) and is implicated in M1-mediated antimycobacterial responses in myeloid cells. L-citrulline, a by-product of NO production, contributes to host defense and generates L-arginine in myeloid cells. In arginase 1-expressing cells, L-arginine can be converted into ornithine, a polyamine precursor that enhances autophagy and antimicrobial protection against Mtb in Kupffer cells. Gamma-aminobutyric acid (GABA), a metabolite and neurotransmitter, activate autophagy to induce antimycobacterial host defenses. This review discusses the recent updates of the functions of the three metabolites in host protection against mycobacterial infection. Understanding the mechanisms by which these metabolites promote host defense will facilitate the development of novel host-directed therapeutics against Mtb and drug-resistant bacteria.

## Introduction

Metabolites function as innate immune effectors against intracellular bacterial infections, including *Mycobacterium tuberculosis* (Mtb), the primary pathogen of human tuberculosis (TB). The roles of several metabolites have been determined in host defense against Mtb infection. A significant advance occurred with identifying itaconate, produced by immunoresponsive gene 1 (Irg1) enzymatic activity, in the growth inhibition of mycobacteria possessing isocitrate lyase (1, 2). A recent discovery (3, 4) regarding the anti-inflammatory function of itaconate points to a role in regulating pathological inflammation during Mtb infection. In addition, an earlier study showed that L-arginine (Arg) metabolism is closely related to bacteriostatic activity in macrophages (5). L-Arg consumption, which is accompanied by nitrite/nitrate production, and L-citrulline exert a fungistatic effect in murine macrophages (5). When produced by arginase-expressing macrophages, L-citrulline activates antimycobacterial responses (6). Ornithine, another metabolite of the L-Arg metabolic pathway, is mainly produced by Kupffer cells in the liver and participates in host defense by activating autophagy (7). Furthermore, γ-aminobutyric acid (GABA), a metabolite and neurotransmitter, activates autophagy to induce innate host defenses against intracellular bacteria, including mycobacteria and salmonella (8).

In recent years, several comprehensive studies have indicated the immunoregulatory functions of these metabolites during host-pathogen interactions in Mtb infection. Here, we focus on the roles and mechanisms by which three metabolites—itaconate, Arg, and GABA—enhance host defenses in macrophages during Mtb infection. Understanding the molecular mechanisms by which metabolites modulate host innate immune pathways will provide therapeutic insight into emerging diseases and human TB.

## Overview of Immunometabolism in Host-Mtb Infections

Macrophages are the principal host phagocytes for Mtb at sites of infection. After phagocytosis by macrophages, Mtb can reside in the phagosomes and circumvent host immune protection by resisting phagolysosomal acidification (9–12). Several macrophage populations are implicated in innate immune defense and infectious pathogenesis, depending on the context (4, 11, 13). Macrophages initiate intracellular innate immune signaling to activate early inflammatory responses following recognition of Mtb and/or its components *via* specific pattern-recognition receptors (10, 12, 14). Macrophages can be categorized into two major types, *i*.*e*., classically activated (M1) and alternatively activated (M2). M1 macrophages exhibit high microbicidal activity and produce proinflammatory cytokines such as tumor necrosis factor (TNF)-α and interleukin (IL)-1β, and inducible nitric oxide synthase (iNOS). By contrast, M2 macrophages participate in tissue repair and produce IL-10, tumor growth factor (TGF)-β, and anti-inflammatory cytokines (15, 16). In addition, macrophages have multiple antimicrobial pathways linking innate immune signaling to various effector mechanisms, including cell-autonomous autophagy pathways. Autophagy activation enhances phagosomal maturation to promote host defense against intracellular mycobacterial infection (10, 12, 17–20). Mtb has multiple strategies for manipulating host innate immune responses and escaping from autophagy to survive inside macrophages (10, 12). A deeper understanding of molecular crosstalks between Mtb and host cells would facilitate the development of new therapeutic strategies against human TB, particularly drug-resistant TB.

Macrophages and other immune cells have distinct metabolic and bioenergetics requirements at different stages of infection (21). Studies involving nonhuman primate TB granulomas showed that M1 polarization is related to a favorable disease outcome (22). During infection, TB granulomas are the active sites of host-Mtb interactions, where Mtb develops mechanisms to resist host protective immunity, and the host localizes the condition (23–26). The alveolar macrophages representing bacterial permissiveness are associated with fatty acid β-oxidation, when compared with glycolytically active interstitial macrophages (27). Metabolic rewiring and epigenetic reprogramming are critical for *M. bovis* bacillus Calmette-Guérin (BCG)-mediated trained immunity, *i*.*e*., long-term immune response to infection or vaccination (28–30). Therefore, the orchestrating immunologic and metabolic responses may determine the outcome of Mtb infection. A general overview of immune metabolic remodeling profiles and the underlying mechanisms are reviewed elsewhere (21, 31, 32) and not discussed here.

## Role of Itaconate During Mycobacterial Infection

Conversion of isocitrate to succinate and glyoxylate by isocitrate lyase is the first step in the glyoxylate shunt in several bacteria, including Mtb (33–35), and is involved in Mtb survival during chronic infection (1, 36). Michelucci et al. showed that itaconic acid (also known as methylenesuccinic acid), produced by Irg1, restricts the growth of bacteria harboring isocitrate lyase, such as Mtb and *Salmonella enterica* (1). In addition, itaconate and itaconyl-CoA target methylcitrate lyase in the methylcitrate cycle and B_12_-dependent methylmalonyl-CoA mutase, respectively, to restrict bacterial growth (1, 37). However, a recent report demonstrates that the Mtb effector Rv2498c, a bifunctional β-hydroxyacyl-CoA lyase, participates in itaconate dissimilation to confer resistance to itaconate (38). Notably, the MtbΔ*rv2498c* strain shows significantly attenuated virulence in a mouse low-dose aerosol infection model (38). Itaconate also has an immunomodulatory function during infection. Irg1, a mitochondrial enzyme for itaconate synthesis, promotes antimicrobial immune responses against Mtb infection by regulating neutrophil-mediated pathological inflammation during infection (39). Indeed, Irg1 suppresses the production of proinflammatory cytokines and reactive oxygen species (ROS). Significantly, treatment of *Irg1* ^-/-^ bone marrow-derived macrophages with a physiologically relevant dose of itaconate (0.25 mM) shifts the transcriptional patterns toward wild-type macrophages (39). Although Irg1-mediated itaconate production is essential for antimycobacterial responses by regulating excessive pulmonary inflammation, it is unclear how Irg1 regulates inflammatory responses in the context of Mtb infection.

**Figure 1:**
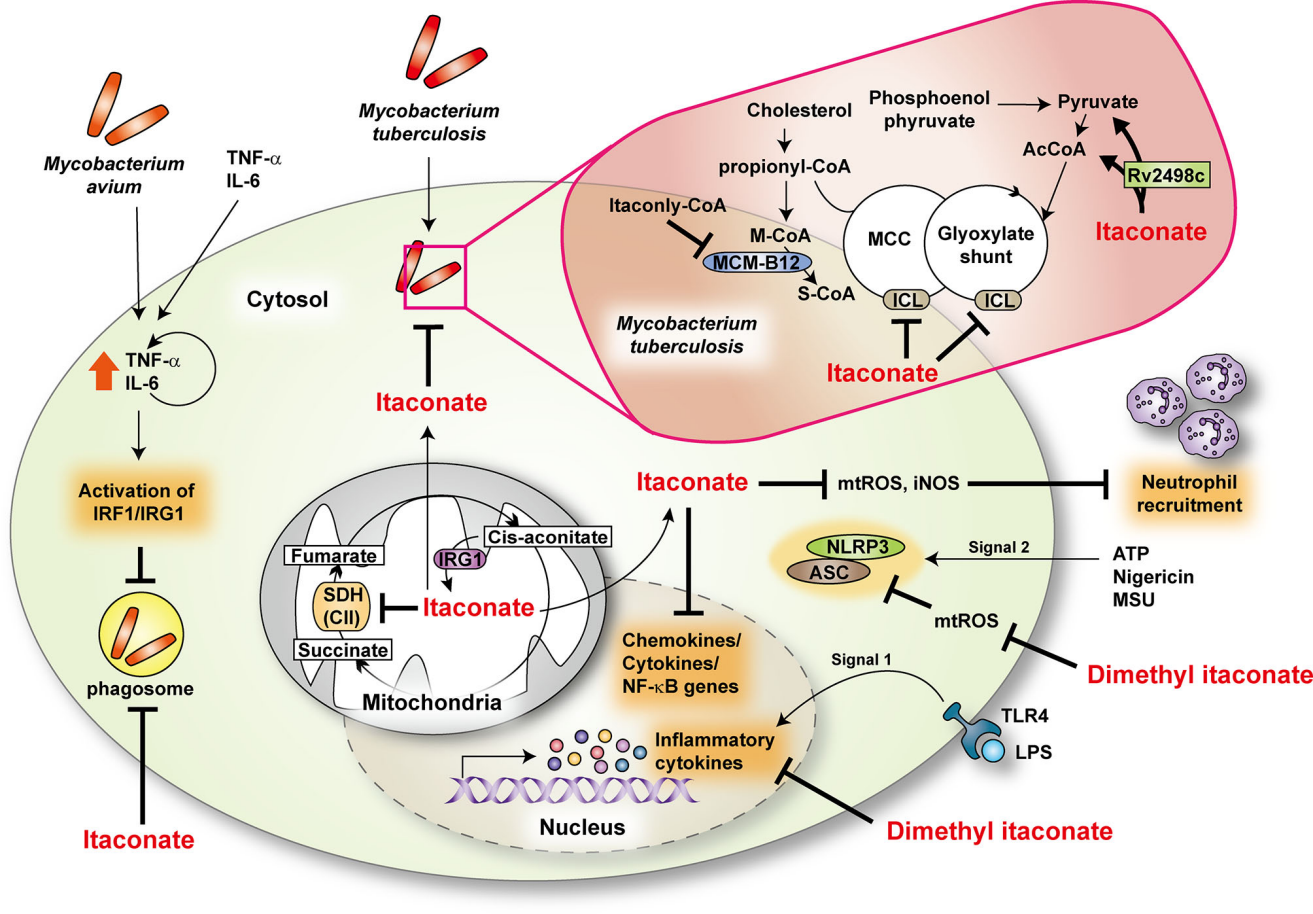
The role of itaconate during mycobacterial infection. Itaconate, produced by immunoresponsive gene 1 (IRG1) from *cis*-aconitate, modulates TCA cycle by regulating succinate dehydrogenase (SDH, as complex II) activity. Dimethyl itaconate regulates the mRNA expression of inflammatory cytokines in response to LPS. In addition, dimethyl itaconate inhibits SDH and decreases the production of mitochondrial ROS (mtROS) in LPS-treated macrophages. It suppresses the expression of NLRP3 and ASC in NLRP3-activating conditions. During Mtb infection, IRG1 and itaconate downregulate inflammatory responses at the transcriptional level and neutrophil recruitment through inhibiting the production of mtROS and inducible nitric oxide synthase (iNOS). In Mtb, itaconate has an antimicrobial activity for methyl citrate lyase (MCL) in the methyl citrate cycle (MCC) and isocitrate lyase (ICL) in glyoxylate shunt, enzymes that are needed for Mtb survival. Moreover, itaconyl-coenzyme A (CoA) targets B_12_-dependent methylmalonyl-CoA mutase (MCM-B12) to restrict bacterial growth. Mtb effector Rv2498c participates in itaconate dissimilation to confer resistance to itaconate. During *M.avium* infection, tumor necrosis factor- α (TNF-α) and interleukin (IL)-6 activate interferon regulatory factor 1 (IRF1)/IRG1 through the autocrine/paracrine signaling pathway. AcCoA, acetyl-coenzyme A; ASC, apoptosis-associated speck-like protein containing a CARD; ATP, adenosine triphosphate; LPS, lipopolysaccharide; M-CoA, methylmalonyl-coenzyme A; MSU, monosodium urate; NLRP3, NLR family pyrin domain containing 3; S-CoA, succinyl-coenzyme A; TLR4, toll-like receptor 4.

Recently, several studies highlighted using the cell-permeable itaconate derivatives to enhance intracellular delivery. Lampropoulou et al. showed that exogenous dimethyl itaconate at physiological doses markedly inhibits *S. typhimurium*-induced IL-1β, nitric oxide (NO), but not TNF-α, in macrophages (40). The underlying mechanisms are intriguing because both NO and IL-1β are crucial components of antimycobacterial immune responses in murine models (41–44). Itaconate treatment also increases the extracellular acidification rate and inhibits succinate dehydrogenase (Sdh), complex II of the mitochondrial electron transport chain, decreasing mitochondrial respiration (40). However, it is unclear whether itaconate-mediated antimycobacterial responses are associated with increased aerobic glycolysis. Moreover, treatment of human primary macrophages with TNF-α and IL-6 suppresses the intracellular growth of *M. avium* through by-stander effects *via* inducing expression of IRG1 (45). Although the endogenous itaconate level is low, direct delivery of itaconate to *M. avium* phagosomes may contribute to antimicrobial responses (45) (**Figure 1**).

To date, the function of itaconate in alveolar macrophages is unclear, although these cells are the first cells that encounter Mtb in the initiation of infection. Future studies are warranted to clarify the role of itaconate in alveolar macrophages during Mtb infection. Recent studies showed that the Irg1 is critically required to control Brucella infection and that dimethyl itaconate has an inhibitory effect against *Brucella* growth (46). Together, these studies reveal an antimicrobial role for itaconate during bacterial infections, including mycobacteria. However, more studies are warranted to clarify the underlying mechanisms of the itaconate functions of various immune cells, including alveolar macrophages, in mycobacterial diseases.

In an inflammation model, itaconate participates in metabolic remodeling in macrophages toward an anti-inflammatory response by activating nuclear factor erythroid 2–related factor 2 (Nrf2) and inhibiting transcription factor Iκβξ-activating transcription factor 3 inflammatory signaling (47, 48). There are controversial findings upon the function of Nrf2 in the context of mycobacterial infection. Nrf2 is critical for host resistance to pulmonary *M. avium* complex infection (49, 50); however, it functions in the antioxidant transcriptional responses that delay early Mtb clearance (51). Future studies are needed to address how Nrf2 signaling is associated with itaconate-mediated protection against mycobacterial infection. Moreover, either 4-octyl itaconate or dimethyl itaconate exerts anti-inflammatory effects and inhibits aerobic glycolysis, thus controlling pathologies related to excessive inflammation (4, 46, 52, 53). Therefore these itaconate derivatives may enhance antimycobacterial responses by controlling pathologic inflammation and immunometabolism during infection. More work is needed to define the molecular mechanisms by which endogenous/exogenous itaconate exerts innate host defenses against mycobacterial diseases.

## Role of Arg Metabolism During Mycobacterial Infection

Arg metabolism promotes antimicrobial responses in myeloid cells by inducing NO and regulating inflammatory responses (54, 55). M1 and M2 macrophages catabolize Arg *via* iNOS and ARG1, respectively. In M1 macrophages, NO synthesis promotes proinflammatory and microbicidal activities against intracellular bacteria (54, 56–58). Although NO plays a critical role in antimycobacterial effect in murine macrophages, its role in human macrophages is still debatable (59). In addition to NO, macrophage anti-Mtb activities induced by L-Arg are dependent, in part, on aerobic glycolysis (60). Moreover, L-Arg synthesis from L-citrulline in myeloid cells contributes to host defense against *M. bovis* BCG and Mtb H37Rv; deficiency of Ass1 or Asl (to eliminate L-Arg synthesis from l-citrulline) increased mycobacterial growth in macrophages and *in vivo* (61). In M2 macrophages, ARG1 expression is critical for synthesizing ornithine, proline, and polyamines, and contributes to wound healing, defense against parasites, and humoral immunity (54, 57). ARG1 elimination in macrophages reduces the bacterial load in the lung during Mtb infection (62). Additionally, Mtb co-infection with helminths such as *Schistosoma mansoni* increases ARG1 expression in macrophages to aggravate lung inflammation and impair anti-Mtb T cell responses (63). Overall, both iNOS- and ARG1-dependent pathways in Arg metabolism have opposite roles in host defense against Mtb infection. The Arg-citrulline metabolic axis may enhance host control, whereas ARG1-mediated Arg metabolism leads to inadequate antimicrobial responses during intracellular bacterial infection.

**Figure 2:**
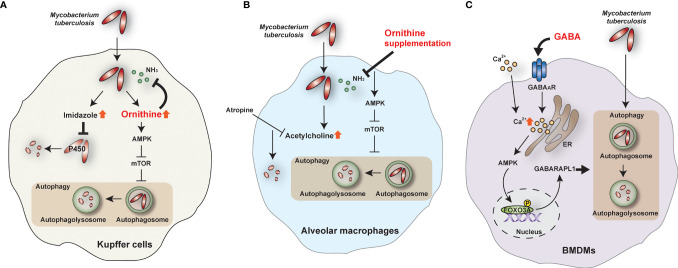
The role of ornithine and GABA during Mtb infection. **(A)** In Kupffer cells, ornithine and imidazole are top-scoring metabolites during Mtb infection. Ornithine restricts Mtb growth by activating the AMPK-mediated autophagy pathway and inhibiting ammonia (NH_3_). On the other hand, imidazole kills Mtb directly by inhibiting cytochrome P450 monooxygenases. **(B)** In alveolar macrophages, ornithine supplementation inhibits NH_3_ and activates autophagy to restrict Mtb growth. In addition, acetylcholine is increased in Mtb-infected alveolar macrophages, and atropine, an acetylcholine antagonist, inhibits Mtb survival. **(C)** GABA induces intracellular calcium (Ca^2+^) influx and activates the autophagy pathway through the AMPK-GABARAPL1 pathway. Autophagy can restrict the intracellular survival of Mtb in bone marrow-derived macrophages (BMDMs). AMPK, AMP-activated protein kinase; FOXO3A, forkhead box O3A; GABA_A_R, GABA A receptor; GABARAPL1, GABA type A receptor-associated protein-like 1; mTOR, mammalian target of rapamycin.

A recent study has identified a novel antimicrobial function of ornithine, an amino acid intermediate produced by ARG1 and ARG2 from Arg metabolism and the urea cycle; and also converts to synthesize proline, polyamines, and citrulline (64, 65). Thandi et al. showed that ornithine is involved in antimycobacterial responses in liver macrophages (7). The authors focused on the liver (7) because of its known role in suppressing Mtb infection (66). Kupffer cells in the liver restrict the growth of Mtb more efficiently than other macrophages, including alveolar macrophages, peritoneal macrophages, and bone marrow-derived macrophages (7). Ornithine promotes Kupffer cell-induced inhibition of intracellular Mtb replication by enhancing autophagy and autolysosome accumulation during Mtb infection (7). Mechanistically, AMP-activated protein kinase (AMPK) is required for ornithine-induced antibacterial autophagy in Kupffer cells during Mtb infection (7). In addition to ornithine, Kupffer cells produce imidazole, which does not induce autophagy but exerts an antimicrobial effect on Mtb by inhibiting mycobacterial cytochrome P450 monooxygenases (7) (**Figure 2A**). However, the mechanism of how ornithine phosphorylates AMPK in Mtb-infected Kupffer cells is unknown. Also, the roles of ornithine and its metabolism at different stages of mycobacterial infection are unclear. Indeed, several types of tumors exhibit increased polyamines, ornithine-related metabolites, leading to transformation and progression (67, 68). In addition, polyamines suppress the intracellular uptake of fluoroquinolones in *M. bovis* BCG and Mtb, thus causing phenotypic antibiotic resistance (69). A deeper understanding of the comprehensive molecular mechanisms by which ornithine, citrulline, and polyamines regulate host antimicrobial responses against Mtb infection would facilitate the development of novel strategies to boost host immune defense against human TB. Targeting the L-Arg-related metabolic network may enable the development of novel vaccines and host-directed therapeutics against human TB.

In contrast to Kupffer cells, alveolar macrophages fail to clear Mtb and facilitate the establishment of Mtb infection through the upregulation of acetylcholine (7). In addition, alveolar macrophages produce higher ammonia $\left({\text{NH}}_{4}^{+}{\text{/NH}}_{3}\right)$ , promoting Mtb growth, compared with Kupffer cells (7). Interestingly, supplementation of ornithine, imidazole, and atropine (acetylcholine inhibitor) promotes Mtb clearance in alveolar macrophages (7) (**Figure 2B**). It is unclear how acetylcholine results in the suppression of Mtb clearance. Given the recent findings that acetylcholine and cholinergic system favor the progression of mycobacterial infection (70), targeting the non-neuronal cholinergic system in the lungs may contribute to the development of new therapeutics against TB.

## Role of GABA During Mycobacterial Infection

GABA is an inhibitory neurotransmitter in the central nervous system and is a metabolite synthesized from glutamic acid by glutamic acid decarboxylase (71–73). Also, GABA is produced in peripheral tissues, including the pancreas, pituitary, testes, gastrointestinal tract, ovaries, placenta, uterus, and adrenal medulla (74, 75). Moreover, peripheral immune cells express GABAergic components—such as type A GABA receptors (GABA_A_R), G-protein-coupled type B receptors (GABA_B_R), and GABA transporters—which modulate GABA biological functions in peripheral tissues and/or cells (76–79). The peripheral GABAergic system plays an essential role in autoimmune and inflammatory diseases like type 1 diabetes, experimental autoimmune encephalomyelitis, collagen-induced arthritis, and dermatitis (77, 80, 81).

GABA signaling activates antimicrobial responses against Mtb and *M. bovis* BCG (8). GABAergic activation *via* GABA_A_R agonists—such as GABA, muscimol, and isoguvacine hydrochloride—induces antibacterial autophagy and phagosomal maturation during Mtb infection. However, GABAergic inhibition suppresses antimicrobial responses during mycobacterial infection. Mechanistically, GABA-induced autophagy activation is mediated by intracellular calcium influx *via* activation of the AMPK-mediated GABA type A receptor-associated protein-like 1 (GABARAPL1) pathway (8) (**Figure 2C**). Indeed, GABA treatment activates AMPK-mediated autophagy and GABA_A_R signaling in intestinal epithelial cells to inhibit enterotoxigenic *Escherichia coli*-induced excessive apoptosis (82). Certainly, AMPK is a crucial metabolic and autophagic regulator and promotes antimicrobial responses against Mtb infection (83–86). However, it is unclear how GABA triggers intracellular calcium influx in peripheral cells to activate AMPK-autophagy pathways. A recent study suggested a molecular framework for GABA-induced Ca^2+^ influx in the context of parasitic infection and immune cell migration (87). In myeloid mononuclear phagocytes such as dendritic cells, glutamate-derived GABA is secreted to trigger GABA_A_R signaling, implicated in Na-K-Cl cotransporters and extracellular Ca^2+^ influx. The result is phagocyte hypermotility and dissemination of the coccidian parasites *Toxoplasma gondii* and *Neospora caninum* (87). Further research is required to identify the role of GABA signaling in different cell types in response to infectious agents.

GABA treatment substantially reduces inflammatory cytokine production in macrophages and lung tissues from infected mice (8). The autocrine or paracrine function of GABA is associated with inhibition of inflammation to ameliorate autoimmune pathologic responses (88, 89). In addition, GABA administration attenuates insulin resistance, obesity-induced adipose tissue macrophage infiltration, and inflammatory responses in subcutaneous inguinal adipose tissues, at least in part *via* GABA_B_R signaling (90). Therefore, GABA signaling pathways can be therapeutic targets for pathologic inflammation during Mtb infection. By contrast, GABA-mediated mammalian target of rapamycin (mTOR) signaling is required for Th17 cell differentiation in the presence of GABA transporter-2 (SLC6A13) deficiency (91). These data suggest a pleiotropic function for GABA in regulating inflammatory responses *via* downstream signaling molecules.

Mtb co-opts GABA as an immune-escape strategy to favor intracellular infection. Mtb can adapt to acidic conditions and oxidative stresses *via* the GABA shunt pathway to reduce NAD^+^ and proton levels (92). In addition, Mtb uses lactate and pyruvate from host cells *via* multiple metabolic pathways, including the GABA shunt (35). Given that GABA promotes host protection against intracellular bacterial infection (8), further research should investigate the molecular mechanisms underlying GABAergic defense in host-pathogen interactions during infection. Pavić et al. revealed that MsGabP, a putative GABA transport protein from *M. smegmatis* and an Mtb homolog, binds GABA and may outcompete the host GABAergic protective system, providing a new target for TB drug development (93). A challenging caveat in developing the immunometabolite-targeted host defensive strategies is the complicated host-mycobacterial relationship to compete for the specific metabolites for their purposes during infection. Further studies are needed to understand better how metabolic communication between Mtb and host cells impact disease outcomes during infection.

## Conclusion

The metabolic interaction between host and mycobacteria is a critical determinant of infection outcomes. However, it is unclear how metabolites and their relationships with other metabolic pathways promote the establishment of chronic infection or control of mycobacteria. In addition, it remains unclear how Mtb manipulates host metabolic fluxes and enzymes to escape immune surveillance and survive intracellularly. The complex interactions between host cells and pathogens *via* metabolic and immune pathways during Mtb infection will be challenging to unravel.

Itaconate and its derivatives exert bactericidal and immuno-modulatory effects critical for antimicrobial defense. Citrulline is a precursor of L-Arg and linked to iNOS-mediated antimycobacterial responses in immune cells; ornithine induces autophagy in Kupffer cells. GABA signaling activates autophagy against intracellular bacteria. Future studies should investigate how these and other immunometabolites influence antimicrobial host defense and pathological inflammation to facilitate a rational design for host-directed therapeutics for mycobacterial infection.

## Author Contributions

JK wrote the manuscript and made the illustrations. E-JP was responsible for reviewing and editing the manuscript. E-KJ designed, supervised, and wrote the manuscript. All authors contributed to the article and approved the submitted version.

## Funding

This work was supported by the National Research Foundation of Korea (NRF) grant funded by the Korea government (MSIT) (No. 2017R1A5A2015385) and by the National Research Foundation of Korea (NRF) grant funded by the Korea government (MSIT) (No.2019R1A2C1087686).

## Conflict of Interest

The authors declare that the research was conducted in the absence of any commercial or financial relationships that could be construed as a potential conflict of interest.

## Publisher’s Note

All claims expressed in this article are solely those of the authors and do not necessarily represent those of their affiliated organizations, or those of the publisher, the editors and the reviewers. Any product that may be evaluated in this article, or claim that may be made by its manufacturer, is not guaranteed or endorsed by the publisher.

## References

[1] Michelucci A Cordes T Ghelfi J Pailot A Reiling N Goldmann O. Immune-Responsive Gene 1 Protein Links Metabolism to Immunity by Catalyzing Itaconic Acid Production. Proc Natl Acad Sci USA (2013) 110:7820–5. doi: 10.1073/pnas.1218599110 PMC3651434 23610393

[2] Zhu X Guo Y Liu Z Yang J Tang H Wang Y. Itaconic Acid Exerts Anti-Inflammatory and Antibacterial Effects *via* Promoting Pentose Phosphate Pathway to Produce ROS. Sci Rep (2021) 11:18173. doi: 10.1038/s41598-021-97352-x 34518559 PMC8438069

[3] Mills EL Ryan DG Prag HA Dikovskaya D Menon D Zaslona Z. Itaconate is an Anti-Inflammatory Metabolite That Activates Nrf2 *via* Alkylation of KEAP1. Nature (2018) 556:113–17. doi: 10.1038/nature25986 PMC6047741 29590092

[4] Liao ST Han C Xu DQ Fu XW Wang JS Kong LY. 4-Octyl Itaconate Inhibits Aerobic Glycolysis by Targeting GAPDH to Exert Anti-Inflammatory Effects. Nat Commun (2019) 10:5091. doi: 10.1038/s41467-019-13078-5 31704924 PMC6841710

[5] Granger DL Hibbs JB Jr. Perfect JR Durack DT. Metabolic Fate of L-Arginine in Relation to Microbiostatic Capability of Murine Macrophages. J Clin Invest (1990) 85:264–73. doi: 10.1172/JCI114422 PMC296414 2404026

[6] Rapovy SM Zhao J Bricker RL Schmidt SM Setchell KD Qualls JE. Differential Requirements for L-Citrulline and L-Arginine During Antimycobacterial Macrophage Activity. J Immunol (2015) 195:3293–300. doi: 10.4049/jimmunol.1500800 PMC6432794 26311904

[7] Sivangala Thandi R Radhakrishnan RK Tripathi D Paidipally P Azad AK Schlesinger LS. Ornithine-A Urea Cycle Metabolite Enhances Autophagy and Controls *Mycobacterium tuberculosis* Infection. Nat Commun (2020) 11:3535. doi: 10.1038/s41467-020-17310-5 32669568 PMC7363810

[8] Kim JK Kim YS Lee HM Jin HS Neupane C Kim S. GABAergic Signaling Linked to Autophagy Enhances Host Protection Against Intracellular Bacterial Infections. Nat Commun (2018) 9:4184. doi: 10.1038/s41467-018-06487-5 30305619 PMC6180030

[9] Vergne I Chua J Singh SB Deretic V. Cell Biology of *Mycobacterium tuberculosis* Phagosome. Annu Rev Cell Dev Biol (2004) 20:367–94. doi: 10.1146/annurev.cellbio.20.010403.114015 15473845

[10] Dey B Bishai WR. Crosstalk Between *Mycobacterium tuberculosis* and the Host Cell. Semin Immunol (2014) 26:486–96. doi: 10.1016/j.smim.2014.09.002 PMC4250340 25303934

[11] Rajaram MV Ni B Dodd CE Schlesinger LS. Macrophage Immunoregulatory Pathways in Tuberculosis. Semin Immunol (2014) 26:471–85. doi: 10.1016/j.smim.2014.09.010 PMC4314327 25453226

[12] Zhai W Wu F Zhang Y Fu Y Liu Z. The Immune Escape Mechanisms of *Mycobacterium tuberculosis* . Int J Mol Sci (2019) 20(2):340. doi: 10.3390/ijms20020340 PMC6359177 30650615

[13] Flynn JL Chan J Lin PL. Macrophages and Control of Granulomatous Inflammation in Tuberculosis. Mucosal Immunol (2011) 4:271–8. doi: 10.1038/mi.2011.14 PMC3311958 21430653

[14] Corleis B Dorhoi A. Early Dynamics of Innate Immunity During Pulmonary Tuberculosis. Immunol Lett (2020) 221:56–60. doi: 10.1016/j.imlet.2020.02.010 32092359

[15] Martinez FO Gordon S. The M1 and M2 Paradigm of Macrophage Activation: Time for Reassessment. F1000Prime Rep (2014) 6:13. doi: 10.12703/P6-13 24669294 PMC3944738

[16] Orecchioni M Ghosheh Y Pramod AB Ley K. Macrophage Polarization: Different Gene Signatures in M1(LPS+) vs. Classically and M2(LPS-) vs. Alternatively Activated Macrophages. Front Immunol (2019) 10:1084. doi: 10.3389/fimmu.2019.01084 31178859 PMC6543837

[17] Gutierrez MG Master SS Singh SB Taylor GA Colombo MI Deretic V. Autophagy is a Defense Mechanism Inhibiting BCG and *Mycobacterium tuberculosis* Survival in Infected Macrophages. Cell (2004) 119:753–66. doi: 10.1016/j.cell.2004.11.038 15607973

[18] Yuk JM Yoshimori T Jo EK. Autophagy and Bacterial Infectious Diseases. Exp Mol Med (2012) 44:99–108. doi: 10.3858/emm.2012.44.2.032 22257885 PMC3296818

[19] Siqueira MDS Ribeiro RM Travassos LH. Autophagy and its Interaction With Intracellular Bacterial Pathogens. Front Immunol (2018) 9:935. doi: 10.3389/fimmu.2018.00935 29875765 PMC5974045

[20] Kim YS Silwal P Kim SY Yoshimori T Jo EK. Autophagy-Activating Strategies to Promote Innate Defense Against Mycobacteria. Exp Mol Med (2019) 51:1–10. doi: 10.1038/s12276-019-0290-7 PMC6906292 31827065

[21] Kumar R Singh P Kolloli A Shi L Bushkin Y Tyagi S. Immunometabolism of Phagocytes During *Mycobacterium tuberculosis* Infection. Front Mol Biosci (2019) 6:105. doi: 10.3389/fmolb.2019.00105 31681793 PMC6803600

[22] Marino S Cilfone NA Mattila JT Linderman JJ Flynn JL Kirschner DE. Macrophage Polarization Drives Granuloma Outcome During *Mycobacterium tuberculosis* Infection. Infect Immun (2015) 83:324–38. doi: 10.1128/IAI.02494-14 PMC4288886 25368116

[23] Saunders BM Britton WJ. Life and Death in the Granuloma: Immunopathology of Tuberculosis. Immunol Cell Biol (2007) 85:103–11. doi: 10.1038/sj.icb.7100027 17213830

[24] Mishra A Surolia A. *Mycobacterium tuberculosis*: Surviving and Indulging in an Unwelcoming Host. IUBMB Life (2018) 70:917–25. doi: 10.1002/iub.1882 30129097

[25] Pagan AJ Ramakrishnan L. The Formation and Function of Granulomas. Annu Rev Immunol (2018) 36:639–65. doi: 10.1146/annurev-immunol-032712-100022 29400999

[26] Russell DG VanderVen BC Lee W Abramovitch RB Kim MJ Homolka S. *Mycobacterium tuberculosis* Wears What it Eats. Cell Host Microbe (2010) 8:68–76. doi: 10.1016/j.chom.2010.06.002 20638643 PMC2929656

[27] Huang L Nazarova EV Tan S Liu Y Russell DG. Growth of *Mycobacterium tuberculosis In Vivo* Segregates With Host Macrophage Metabolism and Ontogeny. J Exp Med (2018) 215:1135–52. doi: 10.1084/jem.20172020 PMC5881470 29500179

[28] Liu Y Liang S Ding R Hou Y Deng F Ma X. BCG-Induced Trained Immunity in Macrophage: Reprograming of Glucose Metabolism. Int Rev Immunol (2020) 39:83–96. doi: 10.1080/08830185.2020.1712379 31933415

[29] Covian C Fernandez-Fierro A Retamal-Diaz A Diaz FE Vasquez AE Lay MK. BCG-Induced Cross-Protection and Development of Trained Immunity: Implication for Vaccine Design. Front Immunol (2019) 10:2806. doi: 10.3389/fimmu.2019.02806 31849980 PMC6896902

[30] Arts RJW Moorlag S Novakovic B Li Y Wang SY Oosting M. BCG Vaccination Protects Against Experimental Viral Infection in Humans Through the Induction of Cytokines Associated With Trained Immunity. Cell Host Microbe (2018) 23:89–100.e5. doi: 10.1016/j.chom.2017.12.010 29324233

[31] Sheedy FJ Divangahi M. Targeting Immunometabolism in Host Defence Against *Mycobacterium tuberculosis* . Immunology (2021) 162:145–59. doi: 10.1111/imm.13276 PMC7808148 33020911

[32] Howard NC Khader SA. Immunometabolism During *Mycobacterium tuberculosis* Infection. Trends Microbiol (2020) 28:832–50. doi: 10.1016/j.tim.2020.04.010 PMC7494650 32409147

[33] Moynihan MM Murkin AS. Cysteine is the General Base That Serves in Catalysis by Isocitrate Lyase and in Mechanism-Based Inhibition by 3-Nitropropionate. Biochemistry (2014) 53:178–87. doi: 10.1021/bi401432t 24354272

[34] Puckett S Trujillo C Wang Z Eoh H Ioerger TR Krieger I. Glyoxylate Detoxification is an Essential Function of Malate Synthase Required for Carbon Assimilation in *Mycobacterium tuberculosis* . Proc Natl Acad Sci USA (2017) 114:E2225–32. doi: 10.1073/pnas.1617655114 PMC5358392 28265055

[35] Serafini A Tan L Horswell S Howell S Greenwood DJ Hunt DM. *Mycobacterium tuberculosis* Requires Glyoxylate Shunt and Reverse Methylcitrate Cycle for Lactate and Pyruvate Metabolism. Mol Microbiol (2019) 112:1284–307. doi: 10.1111/mmi.14362 PMC6851703 31389636

[36] Kratky M Vinsova J. Advances in Mycobacterial Isocitrate Lyase Targeting and Inhibitors. Curr Med Chem (2012) 19:6126–37. doi: 10.2174/092986712804485782 23092127

[37] Ruetz M Campanello GC Purchal M Shen H McDevitt L Gouda H. Itaconyl-CoA Forms a Stable Biradical in Methylmalonyl-CoA Mutase and Derails Its Activity and Repair. Science (2019) 366:589–93. doi: 10.1126/science.aay0934 PMC7070230 31672889

[38] Wang H Fedorov AA Fedorov EV Hunt DM Rodgers A Douglas HL. An Essential Bifunctional Enzyme in *Mycobacterium tuberculosis* for Itaconate Dissimilation and Leucine Catabolism. Proc Natl Acad Sci USA (2019) 116:15907–13. doi: 10.1073/pnas.1906606116 PMC6689899 31320588

[39] Nair S Huynh JP Lampropoulou V Loginicheva E Esaulova E Gounder AP. Irg1 Expression in Myeloid Cells Prevents Immunopathology During *M. tuberculosis* Infection. J Exp Med (2018) 215:1035–45. doi: 10.1084/jem.20180118 PMC5881474 29511063

[40] Lampropoulou V Sergushichev A Bambouskova M Nair S Vincent EE Loginicheva E. Itaconate Links Inhibition of Succinate Dehydrogenase With Macrophage Metabolic Remodeling and Regulation of Inflammation. Cell Metab (2016) 24:158–66. doi: 10.1016/j.cmet.2016.06.004 PMC5108454 27374498

[41] Yang K Wu YJ Xie HP Li M Ming SQ Li LY. Macrophage-Mediated Inflammatory Response Decreases Mycobacterial Survival in Mouse MSCs by Augmenting NO Production. Sci Rep (2016) 6:27326. doi: 10.1038/srep27326 27251437 PMC4890015

[42] Yamada H Mizumo S Horai R Iwakura Y Sugawara I. Protective Role of Interleukin-1 in Mycobacterial Infection in IL-1 Alpha/Beta Double-Knockout Mice. Lab Invest (2000) 80:759–67. doi: 10.1038/labinvest.3780079 10830786

[43] Chao WC Yen CL Hsieh CY Huang YF Tseng YL Nigrovic PA. Mycobacterial Infection Induces Higher Interleukin-1beta and Dysregulated Lung Inflammation in Mice With Defective Leukocyte NADPH Oxidase. PLoS One (2017) 12:e0189453. doi: 10.1371/journal.pone.0189453 29228045 PMC5724816

[44] Jamaati H Mortaz E Pajouhi Z Folkerts G Movassaghi M Moloudizargari M. Nitric Oxide in the Pathogenesis and Treatment of Tuberculosis. Front Microbiol (2017) 8:2008. doi: 10.3389/fmicb.2017.02008 29085351 PMC5649180

[45] Gidon A Louet C Rost LM Bruheim P Flo TH. The Tumor Necrosis Factor Alpha and Interleukin 6 Auto-Paracrine Signaling Loop Controls *Mycobacterium avium* Infection *via* Induction of IRF1/IRG1 in Human Primary Macrophages. mBio (2021) 12:e0212121. doi: 10.1128/mBio.02121-21 34607464 PMC8546851

[46] Demars A Vitali A Comein A Carlier E Azouz A Goriely S. Aconitate Decarboxylase 1 Participates in the Control of Pulmonary *Brucella* Infection in Mice. PLoS Pathog (2021) 17:e1009887. doi: 10.1371/journal.ppat.1009887 34525130 PMC8443048

[47] Bambouskova M Gorvel L Lampropoulou V Sergushichev A Loginicheva E Johnson K. Electrophilic Properties of Itaconate and Derivatives Regulate the IkappaBzeta-ATF3 Inflammatory Axis. Nature (2018) 556:501–04. doi: 10.1038/s41586-018-0052-z PMC6037913 29670287

[48] O’Neill LAJ Artyomov MN. Itaconate: The Poster Child of Metabolic Reprogramming in Macrophage Function. Nat Rev Immunol (2019) 19:273–81. doi: 10.1038/s41577-019-0128-5 30705422

[49] Nakajima M Matsuyama M Kawaguchi M Kiwamoto T Matsuno Y Morishima Y. Nrf2 Regulates Granuloma Formation and Macrophage Activation During *Mycobacterium avium* Infection *via* Mediating Nramp1 and HO-1 Expressions. mBio (2021) 12(1):e01947–20. doi: 10.1128/mBio.01947-20 PMC7885113 33563837

[50] Matsuyama M Nonaka M Nakajima M Morishima Y Ishii Y Hizawa N. The Role of NRF2 in Mycobacterial Infection. Antioxidants (2021) 10:1861. doi: 10.3390/antiox10121861 34942964 PMC8699052

[51] Rothchild AC Olson GS Nemeth J Amon LM Mai D Gold ES. Alveolar Macrophages Generate a Noncanonical NRF2-Driven Transcriptional Response to *Mycobacterium tuberculosis In Vivo* . Sci Immunol (2019) 4(37):eaaw6693. doi: 10.1126/sciimmunol.aaw6693 31350281 PMC6910245

[52] Diskin C Zotta A Corcoran SE Tyrrell VJ Zaslona Z O’Donnell VB. 4-Octyl-Itaconate and Dimethyl Fumarate Inhibit COX2 Expression and Prostaglandin Production in Macrophages. J Immunol (2021) 207:2561–69. doi: 10.4049/jimmunol.2100488 PMC7613254 34635585

[53] Nakkala JR Yao Y Zhai Z Duan Y Zhang D Mao Z. Dimethyl Itaconate-Loaded Nanofibers Rewrite Macrophage Polarization, Reduce Inflammation, and Enhance Repair of Myocardic Infarction. Small (2021) 17:e2006992. doi: 10.1002/smll.202006992 33719217

[54] Marti ILAA Reith W. Arginine-Dependent Immune Responses. Cell Mol Life Sci (2021) 78:5303–24. doi: 10.1007/s00018-021-03828-4 PMC8257534 34037806

[55] Rodriguez PC Ochoa AC Al-Khami AA. Arginine Metabolism in Myeloid Cells Shapes Innate and Adaptive Immunity. Front Immunol (2017) 8:93. doi: 10.3389/fimmu.2017.00093 28223985 PMC5293781

[56] Das P Lahiri A Lahiri A Chakravortty D. Modulation of the Arginase Pathway in the Context of Microbial Pathogenesis: A Metabolic Enzyme Moonlighting as an Immune Modulator. PLoS Pathog (2010) 6:e1000899. doi: 10.1371/journal.ppat.1000899 20585552 PMC2887468

[57] Munder M. Arginase: An Emerging Key Player in the Mammalian Immune System. Br J Pharmacol (2009) 158:638–51. doi: 10.1111/j.1476-5381.2009.00291.x PMC2765586 19764983

[58] Morris SM Jr. Arginine: Master and Commander in Innate Immune Responses. Sci Signal (2010) 3:pe27. doi: 10.1126/scisignal.3135pe27 20716762

[59] Thomas AC Mattila JT. "Of Mice and Men": Arginine Metabolism in Macrophages. Front Immunol (2014) 5:479. doi: 10.3389/fimmu.2014.00479 25339954 PMC4188127

[60] McKell MC Crowther RR Schmidt SM Robillard MC Cantrell R Lehn MA. Promotion of Anti-Tuberculosis Macrophage Activity by L-Arginine in the Absence of Nitric Oxide. Front Immunol (2021) 12:653571. doi: 10.3389/fimmu.2021.653571 34054815 PMC8160513

[61] Lange SM McKell MC Schmidt SM Zhao J Crowther RR Green LC. L-Arginine Synthesis From L-Citrulline in Myeloid Cells Drives Host Defense Against Mycobacteria *In Vivo* . J Immunol (2019) 202:1747–54. doi: 10.4049/jimmunol.1801569 PMC6401247 30710047

[62] El Kasmi KC Qualls JE Pesce JT Smith AM Thompson RW Henao-Tamayo M. Toll-Like Receptor-Induced Arginase 1 in Macrophages Thwarts Effective Immunity Against Intracellular Pathogens. Nat Immunol (2008) 9:1399–406. doi: 10.1038/ni.1671 PMC2584974 18978793

[63] Monin L Griffiths KL Lam WY Gopal R Kang DD Ahmed M. Helminth-Induced Arginase-1 Exacerbates Lung Inflammation and Disease Severity in Tuberculosis. J Clin Invest (2015) 125:4699–713. doi: 10.1172/JCI77378 PMC4665786 26571397

[64] Muthukumaran Sivashanmugam Jaidev J Umashankar V Sulochana KN. Ornithine and Its Role in Metabolic Diseases: An Appraisal. BioMed Pharmacother (2017) 86:185–94. doi: 10.1016/j.biopha.2016.12.024 27978498

[65] Clemente GS van Waarde A Antunes IF Domling A Elsinga PH. Arginase as a Potential Biomarker of Disease Progression: A Molecular Imaging Perspective. Int J Mol Sci (2020) 21(15):5291. doi: 10.3390/ijms21155291 PMC7432485 32722521

[66] Chong VH. Hepatobiliary Tuberculosis: A Review of Presentations and Outcomes. South Med J (2008) 101:356–61. doi: 10.1097/SMJ.0b013e318164ddbb 18360350

[67] Casero RA Jr. Murray Stewart T Pegg AE. Polyamine Metabolism and Cancer: Treatments, Challenges and Opportunities. Nat Rev Cancer (2018) 18:681–95. doi: 10.1038/s41568-018-0050-3 PMC6487480 30181570

[68] Hesterberg RS Cleveland JL Epling-Burnette PK. Role of Polyamines in Immune Cell Functions. Med Sci (Basel) (2018) 6(1):22. doi: 10.3390/medsci6010022 PMC5872179 29517999

[69] Sarathy JP Lee E Dartois V. Polyamines Inhibit Porin-Mediated Fluoroquinolone Uptake in Mycobacteria. PLoS One (2013) 8:e65806. doi: 10.1371/journal.pone.0065806 23755283 PMC3670895

[70] Islas-Weinstein L Marquina-Castillo B Mata-Espinosa D Paredes-Gonzalez IS Chavez J Balboa L. The Cholinergic System Contributes to the Immunopathological Progression of Experimental Pulmonary Tuberculosis. Front Immunol (2020) 11:581911. doi: 10.3389/fimmu.2020.581911 33679685 PMC7930380

[71] Buddhala C Hsu CC Wu JY. A Novel Mechanism for GABA Synthesis and Packaging Into Synaptic Vesicles. Neurochem Int (2009) 55:9–12. doi: 10.1016/j.neuint.2009.01.020 19428801

[72] Serrano-Regal MP Bayon-Cordero L Ordaz RP Garay E Limon A Arellano RO. Expression and Function of GABA Receptors in Myelinating Cells. Front Cell Neurosci (2020) 14:256. doi: 10.3389/fncel.2020.00256 32973453 PMC7472887

[73] Baizabal-Carvallo JF. The Neurological Syndromes Associated With Glutamic Acid Decarboxylase Antibodies. J Autoimmun (2019) 101:35–47. doi: 10.1016/j.jaut.2019.04.007 31000408

[74] Gladkevich A Korf J Hakobyan VP Melkonyan KV. The Peripheral GABAergic System as a Target in Endocrine Disorders. Auton Neurosci (2006) 124:1–8. doi: 10.1016/j.autneu.2005.11.002 16338174

[75] Everington EA Gibbard AG Swinny JD Seifi M. Molecular Characterization of GABA-A Receptor Subunit Diversity Within Major Peripheral Organs and Their Plasticity in Response to Early Life Psychosocial Stress. Front Mol Neurosci (2018) 11:18. doi: 10.3389/fnmol.2018.00018 29467616 PMC5807923

[76] Jin Z Mendu SK Birnir B. GABA is an Effective Immunomodulatory Molecule. Amino Acids (2013) 45:87–94. doi: 10.1007/s00726-011-1193-7 22160261 PMC3680704

[77] Prud’homme GJ Glinka Y Wang Q. Immunological GABAergic Interactions and Therapeutic Applications in Autoimmune Diseases. Autoimmun Rev (2015) 14:1048–56. doi: 10.1016/j.autrev.2015.07.011 26226414

[78] Wang C Hao H He K An Y Pu Z Gamper N. Neuropathic Injury-Induced Plasticity of GABAergic System in Peripheral Sensory Ganglia. Front Pharmacol (2021) 12:702218. doi: 10.3389/fphar.2021.702218 34385921 PMC8354334

[79] Malomouzh A Ilyin V Nikolsky E. Components of the GABAergic Signaling in the Peripheral Cholinergic Synapses of Vertebrates: A Review. Amino Acids (2019) 51:1093–102. doi: 10.1007/s00726-019-02754-x 31236726

[80] Siggs OM Berger M Krebs P Arnold CN Eidenschenk C Huber C. A Mutation of Ikbkg Causes Immune Deficiency Without Impairing Degradation of IkappaB Alpha. Proc Natl Acad Sci USA (2010) 107:3046–51. doi: 10.1073/pnas.0915098107 PMC2840324 20133626

[81] Bajic SS Dokic J Dinic M Tomic S Popovic N Brdaric E. GABA Potentiate the Immunoregulatory Effects of *Lactobacillus Brevis* BGZLS10-17 *via* ATG5-Dependent Autophagy *In Vitro* . Sci Rep (2020) 10:1347. doi: 10.1038/s41598-020-58177-2 31992761 PMC6987229

[82] Xia Y Chen S Zhao Y Chen S Huang R Zhu G. GABA Attenuates ETEC-Induced Intestinal Epithelial Cell Apoptosis Involving GABAAR Signaling and the AMPK-Autophagy Pathway. Food Funct (2019) 10:7509–22. doi: 10.1039/c9fo01863h 31670355

[83] Paik S Jo EK. An Interplay Between Autophagy and Immunometabolism for Host Defense Against Mycobacterial Infection. Front Immunol (2020) 11:603951. doi: 10.3389/fimmu.2020.603951 33262773 PMC7688515

[84] Jo EK Silwal P Yuk JM. AMPK-Targeted Effector Networks in Mycobacterial Infection. Front Microbiol (2019) 10:520. doi: 10.3389/fmicb.2019.00520 30930886 PMC6429987

[85] Cheng CY Bohme J Singhal A. Metabolic Energy Sensors as Targets for Designing Host-Directed Therapies for Tuberculosis. J Leukocyte Biol (2018) 103:215–23. doi: 10.1189/jlb.4MR0617-226R 28951420

[86] Jia JY Bissa B Brecht L Allers L Choi SW Gu YX. AMPK, a Regulator of Metabolism and Autophagy, is Activated by Lysosomal Damage *via* a Novel Galectin-Directed Ubiquitin Signal Transduction System. Mol Cell (2020) 77:951–69.e9. doi: 10.1016/j.molcel.2019.12.028 PMC7785494 31995728

[87] Bhandage AK Olivera GC Kanatani S Thompson E Lore K Varas-Godoy M. A Motogenic GABAergic System of Mononuclear Phagocytes Facilitates Dissemination of Coccidian Parasites. Elife (2020) 9:e60528. doi: 10.7554/eLife.60528 33179597 PMC7685707

[88] Bhat R Axtell R Mitra A Miranda M Lock C Tsien RW. Inhibitory Role for GABA in Autoimmune Inflammation. Proc Natl Acad Sci USA (2010) 107:2580–5. doi: 10.1073/pnas.0915139107 PMC2823917 20133656

[89] Nguyen L Malgrange B Breuskin I Bettendorff L Moonen G Belachew S. Autocrine/paracrine Activation of the GABA(A) Receptor Inhibits the Proliferation of Neurogenic Polysialylated Neural Cell Adhesion Molecule-Positive (PSA-NCAM+) Precursor Cells From Postnatal Striatum. J Neurosci (2003) 23:3278–94. doi: 10.1523/JNEUROSCI.23-08-03278.2003 PMC6742317 12716935

[90] Hwang I Jo K Shin KC Kim JI Ji Y Park YJ. GABA-Stimulated Adipose-Derived Stem Cells Suppress Subcutaneous Adipose Inflammation in Obesity. Proc Natl Acad Sci USA (2019) 116:11936–45. doi: 10.1073/pnas.1822067116 PMC6575165 31160440

[91] Ren W Liao Y Ding X Jiang Y Yan J Xia Y. Slc6a13 Deficiency Promotes Th17 Responses During Intestinal Bacterial Infection. Mucosal Immunol (2019) 12:531–44. doi: 10.1038/s41385-018-0111-7 30523310

[92] Rizvi A Shankar A Chatterjee A More TH Bose T Dutta A. Rewiring of Metabolic Network in *Mycobacterium tuberculosis* During Adaptation to Different Stresses. Front Microbiol (2019) 10:2417. doi: 10.3389/fmicb.2019.02417 31736886 PMC6828651

[93] Pavic A Ji Y Serafini A Garza-Garcia A McPhillie MJ Holmes AOM. Functional Characterization of the Gamma-Aminobutyric Acid Transporter From *Mycobacterium smegmatis* Mc(2) 155 Reveals Sodium-Driven GABA Transport. J Bacteriol (2021) 203(4):e00642–20. doi: 10.1128/JB.00642-20 PMC7847548 33288625

